# Spatially resolved immune exhaustion within the alloreactive microenvironment predicts liver transplant rejection

**DOI:** 10.21203/rs.3.rs-3044385/v1

**Published:** 2023-07-03

**Authors:** Arianna Barbetta, Brittany Rocque, Sarah Bangerth, Kelly Street, Carly Weaver, Shefali Chopra, Janet Kim, Linda Sher, Brice Gaudilliere, Omid Akbari, Rohit Kohli, Juliet Emamaullee

**Affiliations:** University of Southern California; University of Southern California; University of Southern California; Keck School of Medicine of U; University of Southern California; University of Southern California; University of Southern California; University of Southern California Keck School of Mdicine; Stanford University; University of Southern California, Keck School of Medicine; University of Southern California; University of Southern California

**Keywords:** Transplant immunology, single-cell analysis, spatial analysis, alloimmunity, mass cytometry, PD1

## Abstract

Allograft rejection is a frequent complication following solid organ transplantation, but defining specific immune subsets mediating alloimmunity has been elusive due to the scarcity of tissue in clinical biopsy specimens. Single cell techniques have emerged as valuable tools for studying mechanisms of disease in complex tissue microenvironments. Here, we developed a highly multiplexed imaging mass cytometry panel, single cell analysis pipeline, and semi-supervised immune cell clustering algorithm to study archival biopsy specimens from 79 liver transplant (LT) recipients with histopathological diagnoses of either no rejection (NR), acute T-cell mediated rejection (TCMR), or chronic rejection (CR). This approach generated a spatially resolved proteomic atlas of 461,816 cells derived from 98 pathologist-selected regions of interest relevant to clinical diagnosis of rejection. We identified 41 distinct cell populations (32 immune and 9 parenchymal cell phenotypes) that defined key elements of the alloimmune microenvironment (AME), identified significant cell-cell interactions, and established higher order cellular neighborhoods. Our analysis revealed that both regulatory (HLA-DR^+^ Treg) and exhausted T-cell phenotypes (PD1^+^CD4^+^ and PD1^+^CD8^+^ T-cells), combined with variations in M2 macrophage polarization, were a unique signature of TCMR. TCMR was further characterized by alterations in cell-to-cell interactions among both exhausted immune subsets and inflammatory populations, with expansion of a CD8 enriched cellular neighborhood comprised of Treg, exhausted T-cell subsets, proliferating CD8^+^ T-cells, and cytotoxic T-cells. These data enabled creation of a predictive model of clinical outcomes using a subset of cell types to differentiate TCMR from NR (AUC = 0.96 ± 0.04) and TCMR from CR (AUC = 0.96 ± 0.06) with high sensitivity and specificity. Collectively, these data provide mechanistic insights into the AME in clinical LT, including a substantial role for immune exhaustion in TCMR with identification of novel targets for more focused immunotherapy in allograft rejection. Our study also offers a conceptual framework for applying spatial proteomics to study immunological diseases in archival clinical specimens.

## INTRODUCTION

T-cell mediated rejection (TCMR) remains the most frequent complication after liver transplantation (LT), occurring within the first six months in up to 35% of adult LT recipients^1–3^. While TCMR is generally responsive to treatment with pulse corticosteroids, adjustment of maintenance immunosuppression regimens is key for preventing future TCMR episodes^4^. Ultimately, up to 10% of patients will develop steroid resistance and have recurrent episodes of TCMR. The diagnosis of TCMR hinges upon histological examination of a core biopsy stained with hematoxylin & eosin by a clinical pathologist using Rejection Activity Index (RAI), a composite score ranging from 0–9 based on severity of portal inflammation, bile duct inflammation, and venous endotheliitis^5,6^. After its inception following a Banff consensus conference in 1995, the RAI has become the gold standard to establish the diagnosis of TCMR and guide treatment strategies in clinical LT. There have been minimal changes in the RAI since it was first introduced, with additional criteria for antibody mediated rejection (AMR), a rare entity in LT, in 2016^6^. In parallel, options for both induction and maintenance immunosuppression in LT have not changed substantially since the 1990s and rely on therapeutics that cause non-specific suppression of entire leukocyte populations. For instance, the two mainstay treatments broadly suppress the T-cell compartment (calcineurin inhibitors), or they function by globally inhibiting both macrophages and T-cells (corticosteroids)^7^. Thus, the absence of specific targeting for TCMR-associated immune subpopulations in LT results in both suboptimal prevention and treatment of TCMR episodes, as well as a variety of unintended, and often severe, adverse medication side effects.

Improving our understanding of the complex alloimmune microenvironment (AME) in clinical LT would enable development of novel, focused, and personalized immunotherapies. Donor-derived antigen presenting cells (APCs) expressing allograft antigen on both MHC I and II can activate host CD8^+^ and CD4^+^ T-cells via the direct pathway, ultimately leading to tissue damage via Fas-FasL or granzyme/perforin production and secretion of pro-inflammatory cytokines^8^. The indirect pathway, which has been implicated in late TCMR, is mediated by recipient APCs infiltrating the allograft over time^8^. However, deeper characterization of graft-infiltrating leukocytes driving TCMR in clinical LT is still needed. When compared to experimental heart and kidney transplantation, small and large animal models of LT are more technically demanding while offering a lower threshold for tolerance induction and thus less opportunity to recapitulate alloimmunity in clinical TCMR^9^. Examination of clinical samples has been limited by the tiny amount of tissue available from a core needle biopsy specimen. The INTERLIVER study examined over 200 clinical LT biopsies using genome microarrays and archetypal analysis, and differentially expressed (DE) genes involving both effector T-cell and injury-related pathways were identified in the small subset of biopsies with TCMR. Supervised and unsupervised molecular classifiers based on the top 30 DE genes had only a modest ability to predict histological TCMR (AUC 0.57 and 0.70, respectively)^10^. A more recent histologic study analyzing post-LT biopsies demonstrated that CD8^+^ T-cells form an immune synapse with APCs, with an association between segregation of CD3 and CD45 molecules on CD8^+^ T-cells, immunosuppression weaning failure, and development of TCMR^11^. Thus, key features driving the intrahepatic alloimmune response, including composition and phenotype of alloreactive T-cell subpopulations and interactions between innate and adaptive cells, remain elusive.

CD4^+^CD25^+^FoxP3^+^ regulatory T-cells (Tregs) have been a central focus in both experimental and clinical LT^12^. Despite substantial evidence that Tregs are central mediators of rejection and immune tolerance, clinical trials designed to expand Treg either via therapeutic intervention or cellular therapies have not yet resulted in positive clinical outcomes^13^. The programmed death 1 (PD1) pathway has also emerged an important physiologic immune checkpoint to maintain peripheral T-cell tolerance and regulate adaptive immune responses particularly during chronic antigen exposure^14^. PD1 can be expressed on both B and T-cell populations, including Tregs upon activation, with constant high expression levels following sustained antigen exposure. The PD1 pathway antagonizes T-cell receptor (TCR) engagement and CD28 co-stimulation signals, attenuating downstream cytokine production, proliferation, cell metabolism and survival; thus, ultimately moderating T-cell activity^15,16^. The role of PD1 signaling in transplantation is not well defined, with preliminary studies on heart and kidney allografts implicating PD1-related signals as markers of allograft rejection^17–22^. However, in a recent study in clinical LT, flow cytometric analysis failed to demonstrate a difference in PD1 expression in allograft-infiltrating T-cells isolated from liver explant (n = 5), rejection (n = 7), and no-rejection liver biopsies (n = 7)^23^. Thus, detailed study of the relationship between different regulatory and inflammatory immune cell populations is critical for defining the important aspects of the AME, optimizing identification of predictive biomarkers of TCMR, and identifying focused targets for immunotherapy.

Here, using multiplexed proteomics-based Imaging Mass Cytometry (IMC) analysis, we developed an immune cell phenotyping and analysis pipeline that enabled granular, single-cell characterization of over 30 discrete immune cell types, with spatial assessment of the AME in a large population of post-LT patients with no rejection (NR), TCMR, and progression to CR. We defined significant cell-to-cell interactions and identified spatial motifs as well as predicted single cell phenotypes associated with TCMR. This approach revealed that within the AME the evolution of the immune response in TCMR was associated with intragraft presence of specific T-cell subpopulations exhibiting an exhaustion phenotype. However, these PD1^+^ T-cells were lost in CR, suggesting an important role as mediators and potential biomarkers of TCMR. Furthermore, we showed that lymphocytes and macrophages are spatially organized into aggregates in which strong interactions among PD1^+^ and effector T-cells exist as well as between CD8^+^ T-cells and specific macrophage subpopulations. Collectively, our data offer a detailed and spatially conscious atlas of immune infiltrates in the liver AME during TCMR episodes that represent putative *in situ* biomarkers of rejection. Our data provide a framework for histologic assessment of complex immune microenvironments at single cell resolution in archival clinical samples, which can inform development of novel clinical assays improving treatment specificity and support development of novel targets for immunotherapy.

## METHODS

This study was approved by the Health Science Campus Institutional Review board of the University of Southern California (HS-18-00708).

### Patients

LT recipients were retrospectively identified using our institutional transplant database. Patients > 18 years at the time of transplant who underwent biopsy of their liver allograft to rule out suspected TCMR or patients with CR undergoing re-transplantation between 1/2000 and 12/2021 met inclusion criteria. Patients were excluded if the histologic diagnosis was associated with reactivation or concurrent viral infection (i.e., Hepatitis C or cytomegalovirus), anatomic causes of graft dysfunction (i.e., vascular stenoses and/or biliary strictures), or advanced fibrosis (bridging fibrosis based on Trichrome staining). Pathology reports were reviewed by a pathologist with expertise in LT to prioritize selection of patients with RAI≥4 for the TCMR group (n = 41 patients, 58 ROIs, median RAI of 5 (Interquartile range (IQR) 5–6)). LT recipients who did not have evidence of rejection on their biopsy (RAI=0) were selected for the NR group (n = 24). The CR patients (n = 14) were identified at the time of re-transplant for CR with histologic confirmation of CR in the explant.

### Clinical Data and Demographics

Demographics and clinical parameters were obtained via comprehensive chart review and included age, sex, ethnicity, race, age at transplant, serum biochemistries, immunosuppression regimens, and all biopsy data including indication for biopsy, timing of biopsy in relation to LT, and pathology reports. Relevant demographic variables are summarized for the cohort in **Extended Data Table 1**. For consistency, RAI score and detailed breakdown of sub-scores were independently reviewed by a liver specialized pathologist. This review showed close agreement with the pathologic evaluation performed at the time of biopsy.

### Imaging Mass Cytometry

Formalin-fixed paraffin embedded (FFPE) tissue sections of liver biopsy specimens or explants (4μm) were selected by the pathologist to identify 1mm^2^ regions of interest (ROI) for IMC acquisition focusing on representative periportal regions of the biopsies used in the clinical assessment of RAI. The SC2 Core Facility at Children’s Hospital-Los Angeles performed all staining and image acquisition for this study. Slides were stained using a custom 22-marker antibody panel. Structural markers included two nuclear intercalator dyes, collagen, CD31 (vascular endothelium), and CK7 (bile ducts). Immune lineage markers included CD3, CD4, CD8, CD20, CD68, CD11b and functional or phenotypic markers included CD279 (PD1), FoxP3, Ki67, and Granzyme B among others (**Extended Data Table 2**). IMC staining was performed using techniques described previously^24^. Ninety-six ROIs (average of 1.2 ROI/patient) were ablated using the Hyperion Imaging System (Standard Biotools) at a power range of 3.5–4.5 with a laser frequency at 200Hz. Data were supplied as .txt and .mcd files for use in segmentation and downstream analyses.

### Image Pre-Processing and Segmentation

Pre-processing steps were completed using the MATLAB package MAUI (MBI Analysis User Interface)^25^. CD68 was used as the basis for channel spillover correction and noise removal and channel aggregate removal steps were implemented individually on each channel. Pre-processing was conducted in three batches by clinical outcome (NR, TCMR, CR), to account for staining background and noise differences between disease states. Cell segmentation was performed using Mesmer (DeepCell) and following the Bodenmiller Steinbock pipeline^26^. Image pre-processing was performed in MATLAB (version R2022b) and Python (version 3.10.8).

### Single Cell Phenotyping

Cell segmentation outputs were loaded into R (version 4.2.2) to perform downstream analysis. Patient ID and clinical group identifiers were added to the Single Cell Experiment object^27^. Data were arcsin transformed using a cofactor of 5 and standardized by channel to account for differences in signal intensities.

Metaclusters were identified using a supervised clustering approach outlined in **Extended Data Table 3**. Labelling accuracy was verified by reviewing concurrent metacluster label and channel expression on tissue sections. Masks were used to visualize cell labels (“cytomapper::plotCells”)^28^. TIFF images were scaled, and channel signals were normalized and visualized individually (“cytomapper::plotPixels”). For each patient, metacluster proportions were calculated using the overall cell count as baseline and statistically compared across clinical groups. Subclustering was performed on the five most relevant immune metaclusters (CD4^+^ T-cells, CD8^+^ T-cells, B cells, macrophages, and monocytes) using a semi-supervised approach. A total of 30 subclusters were identified, leading to a final 32 immune clusters and 9 non-immune clusters in the overall dataset. Dimensionality reduction was performed using t-Distributed Stochastic Neighbor Embedding (t-SNE) to visualize meta- and subclusters by clinical outcome^29^. T-SNE was also used to visualize possible batch effects between patients. Batch correction was performed using the mutual nearest neighbors (MNN) method, but ultimately not used for downstream analysis to avoid possibly also eliminating biological differences present in the data^30^.

### Trajectory Inference

To investigate whether cell phenotypes identified via IMC represented a pseudotemporal evolution of the alloimmune microenvironment in LT rejection, we performed trajectory inference on each metacluster. Metacluster-specific dimensionality reduction was performed by Uniform Manifold Approximation and Projection (UMAP)^31^. Trajectories were identified by Slingshot, an algorithm that can model branching lineages in single-cell data^32^. To ensure proper orientation of each trajectory, a coarse clustering was performed using k-means (k = 2, except for CD8^+^ T-cells where k = 5) and the cluster with the highest proportion of cells from NR samples was set as the initial cluster.

### Spatial Analysis

The k-nearest neighbor approach (k = 10) was used to create the cell-cell interaction graph, which was visualized on tissue using the “imcRtools::plotSpatial” function^26^. Neighborhood analysis (“imcRtools::testInteractions”) was implemented on each clinical subset to analyze pairwise interactions between metaclusters and between subclusters and to compare differences across clinical outcomes. Cell-cell interactions were calculated using permutation testing (1,000 permutations, α=0.01) to determine whether cell types interact more (attraction) or less (avoidance) frequently than random permutations. Graph network analysis using igraph was used to visualize subcluster interactions^33^.

Cellular neighborhood analysis was implemented (“imcRtools::aggregateNeighbors”) using the constructed k-nearest neighbor spatial graph (k = 10). Cells were re-clustered based on the cell types in their direct spatial neighborhood to obtain 9 cellular neighborhoods (CN). Cell type abundance of each CN was visualized on a heatmap to aid CN annotation. For each patient, CN proportions were calculated, visualized, and statistically tested to detect any differences across clinical group. CNs were also visualized on the tissue to detect any visual differences in spatial composition across clinical group.

Due to the spatial relevance of TCMR infiltrates to the vascular endothelium (based off clinical Banff criteria RAI scoring), the median distance of each cell type (meta- and subclusters) to endothelial cells was calculated and compared across clinical groups.

### Predictive Modeling

Logistic LASSO (least absolute shrinkage and selection operator) regression was used to build predictive models of NR vs TCMR, TCMR vs CR, and NR vs CR. LASSO is a shrinkage method that aids in feature selection and avoids overfitting. LASSO adds an L1 regularization term (sum of absolute values of the coefficients) so that the selected coefficients minimize the loss function $L(\beta )=∥y-X\beta {∥}^{2}+\lambda_{1}∥\beta {∥}_{1}$, where y is the vector of the binary clinical outcome, X is the feature matrix, β is the vector of coefficients, and $\lambda_{1}$ is the regularization coefficient. A 5-fold cross-validation (CV) technique was used to find the optimal $\lambda_{1}$ value. For each comparison, model building was done using those cell types found to be statistically significant in the pairwise comparisons as input. Bootstrapping, a sampling with replacement technique, was implemented to rank the importance of all features (5,000 iterations). In each iteration, logistic LASSO regression was implemented on a subset of the data and non-zero coefficients were stored. Variable frequency was determined, and variables with ≥ 50% frequency were selected for the final model. To evaluate model performance, data was cross validated by randomly splitting into training and validation sets at a 75/25 ratio with 1,000 iterations. In each iteration, the model was trained on the training set using the features identified during bootstrapping. Clinical outcome was then predicted on the validation set and stored alongside performance metrics (sensitivity, specificity, accuracy, and area under the curve [AUC]). Final model coefficients were obtained by averaging all coefficients. Final model performance was calculated using the evaluation metrics obtained from all iterations (mean ± SD). Receiver operating characteristic (ROC) curve was calculated using the median prediction of each patient. Correlation between the median prediction and actual clinical outcome was calculated using Spearman correlation and significance was tested using the Wilcoxon Rank-Sum test.

### nCounter Transcriptomic and TCR Expression Analysis

A subset of representative tissue samples (4 NR and 4 TCMR) was selected, and at least five 5μm FFPE sections per block were combined for RNA extraction using the Rneasy Kit (Qiagen). Extracted RNA was quantified using the NanoDrop system (Thermo Fisher Scientific), and 200 ng of total RNA was used for gene expression analysis. Samples were processed using the nCounter Nanostring platform and the PanCancer Immune Profiling and T-cell repertoire panels according to the manufacturer’s guidelines (NanoString Technologies). Raw counts were normalized using internal positive standards and housekeeping genes with the nSolver Analysis 4.0 and Advanced Analysis 2.0 software (NanoString Technologies). Expression of scaled log2 gene counts were visualized using heatmaps to determine expression differences between NR and TCMR samples. Raw counts of TRAV, TRBV, and TRGV and TRDV genes were expressed as a proportion among total TRAV, TRBV, or TRGV and TRDV gene counts, respectively, for each patient and normalized using the median value from the healthy control group. Publicly available data from a study of 6 patients who underwent IL-2 therapy and subsequently had rejection episodes within 6 months post treatment was used to validate some of these findings^34^. The fold-change in mean gene expression between NR and TCMR as well as baseline and 4-weeks post treatment was compared to show similarity in gene upregulation.

### Statistical Analysis

The Shapiro-Wilk test was used to test for normality. One-way ANOVA and two-sample t-test were used to analyze parametric data. Kruskal-Wallis and Wilcoxon Rank-Sum tests were used to analyze non-parametric data. P-values were corrected for multiple testing using the Holm method. Number of cells for each meta- and subcluster were reported as median [Q1, Q3]. A 0.05 p-value cut-off was used throughout the analysis to determine statistical significance. All statistical tests were carried out in R (version 4.2.2).

## RESULTS

### Major cell types and proportions in liver allografts with and without rejection

**Figures 1:**
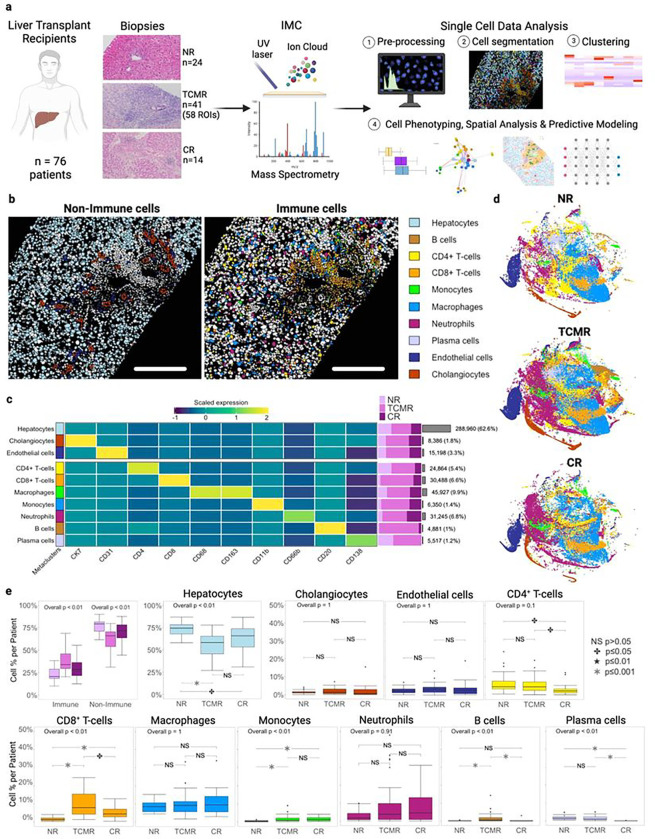
Single cell proteomic atlas of the global cellular composition in liver allografts using IMC. **a.** Schematic figure of IMC workflow starting with case selection of biopsies including 96 specimens from 76 patients across clinical groups (no rejection (NR) n = 24, T-cell mediated rejection (TCMR) n = 41, and chronic rejection (CR) n = 14). Tissue specimens were stained with our 22-Marker IMC panel and images were acquired. Images were pre-processed and then segmented to generate masks and a single cell expression matrix dataset. Downstream phenotypic analysis using a semi-supervised clustering approach and spatial analysis was performed on the dataset containing 461,816 cells. **b**. Representative visualization of cell masks colored by cell population in non-immune and immune populations in TCMR. Scale bar = 190mm. Cell population or metacluster colors from the legend are consistent throughout the figure. **c**. Heatmap showing scaled marker expression within our 10 major metaclusters with purple bars with relative proportion of which clinical group contributed to the metacluster. Gray bars depict total cell number and percent composition of that population across the entire dataset. **d**. t-SNE visualization showing cell metaclusters (excluding hepatocytes for ease of visualizing the less abundant metaclusters) separated by clinical group. **e**. Boxplots representing the relative proportions of metaclusters across clinical groups with statistical comparison of each population as a proportion of that cell type per patient. TCMR and CR showed a greater proportion of immune cells compared to NR. Among the three clinical groups, different cell proportions were observed in CD4^+^ and CD8^+^ T-cells, B-cells, Monocytes and Plasma cells compartments. P values are based on Kruskal-Wallis test and are corrected for multiple tests.

We applied IMC to 24 NR liver core biopsies, 41 biopsies with proven TCMR, and 14 CR samples using our customized analysis pipeline (Fig. 1a). By segmenting the acquired 96 multiplexed images, we generated a single cell atlas of the AME containing a total of 461,816 cells (average 4,811 ± 2,291 cell/ROI) which were classified into 10 main cell populations or ‘metaclusters’. We evaluated raw image signals, post-segmentation dimensionality reduction (t-SNE) of individual markers, immune metaclusters by patient, and difference in mean fold change expression of all markers among the three clinical groups. (**Extended Data** Fig. 1). We first projected metaclusters onto tissue sections, separating out non-immune metaclusters (hepatocytes, cholangiocytes, endothelial cells) and immune metaclusters (CD4^+^ T-cells, CD8^+^ T-cells, macrophages, monocytes, neutrophils, B cells and plasma cells, Fig. 1b). We then quantified the number of cells within each metacluster and evaluated scaled marker expression of lineage markers (Fig. 1c). Hepatocytes were the most common non-immune cell type, representing 62.6% of all cells identified, while macrophages were the most common immune cell type, representing 9.9% of all cells identified. Rare populations were also identified, including cholangiocytes (1.8% of all cells) and B cells (1% of all cells). Next, t-SNE was used to visualize differences in cell metaclusters between clinical groups (Fig. 1d). Proportions of immune and non-immune populations were examined and compared between clinical groups. By looking at the expression of Ki67 and HLADR within non-immune cell populations, we identified three different subclusters of hepatocytes, cholangiocytes, and endothelial cells (**Extended Data** Fig. 2). Proliferating hepatocytes (Ki67^+^ hepatocytes) and HLADR^+^ hepatocytes presented a different distribution across the three groups, with a greater proportion of proliferating hepatocytes in TCMR (p < 0.01) and HLA-DR + hepatocytes in both TCMR and CR when compared to NR (p < 0.01) (**Extended Data** Fig. 2d-e). Similarly, the percentage of HLADR^+^ cholangiocytes differed across the three clinical groups, with a greater percentage of HLADR^+^ cholangiocytes in both TCMR and CR compared to NR (p = 0.01, **Extended Data** Fig. 2i-j). While MHC II molecules are constitutively expressed on human cholangiocytes, the inflammatory state of several diseases including primary biliary cirrhosis, primary sclerosing cholangitis, graft versus host disease, and even liver TCMR has been associated with MHC II overexpression on cholangiocytes, which may function as APCs in the liver^35,36^. Within immune metaclusters, there was an increase in CD8^+^ T-cells between NR and TCMR as well as NR and CR (p < 0.01) with a subtle increase in monocytes from NR to TCMR (p < 0.01, Fig. 1e). Despite macrophages being the most common immune metacluster, which is consistent with their pivotal role in regulating liver immune function, there were no differences in abundance between clinical groups (Fig. 1e, **Extended Data** Fig. 1d)^37,38^.

#### Evaluation of T-cell and Macrophage subpopulations in acute liver allograft rejection shows expansion of exhausted phenotypes.

To uncover the various cell subpopulations and potentially important cell phenotypes within metaclusters, we employed a semi-supervised clustering approach on the CD4^+^, CD8^+^, B cell, macrophage, and monocyte immune metaclusters.

**Figure 2:**
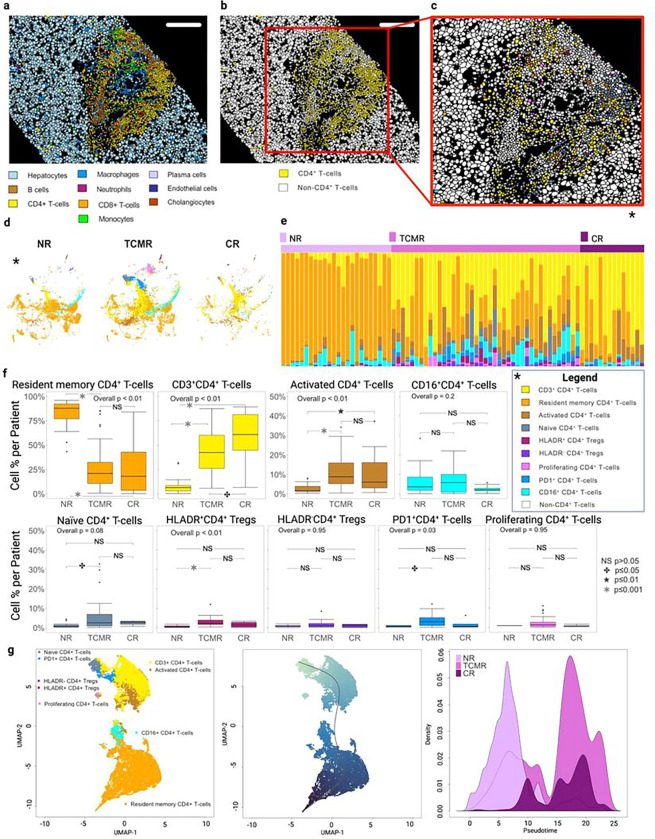
TCMR is uniquely characterized by expansion of Treg and PD1+CD4+ Tcells. **a**. Visualization of cell masks colored by the 10 main metaclusters on representative TCMR tissue section (scale bar = 180mm). **b**. Plot of the same TCMR tissue section with yellow coloring indicating location of CD4^+^ T-cells within the representative core biopsy. **c**. Zoom panel highlighting CD4+ T-Cells colored by cell subpopulation (see color key legend). Subpopulations were identified using unsupervised clustering within the CD4+ T-cell metacluster, which comprised 24,864 cells, using the expression values from markers CD28, CD16, CD11b, CD45, CD4, CD279 (PD1), FoxP3, Ki67 CD3, and HLADR. Nine unique supopulations emerged from this analysis including: Resident Memory CD4^+^ T-cells, CD3^+^CD4^+^ T-cells, Activated (HLADR^hi^) CD4^+^ T-cells, CD16^+^CD4^+^ T-cells, Naïve CD4^+^ T-cells, HLADR^+^CD4^+^ Tregs, HLADR^−^CD4^+^ Tregs, PD1^+^CD4^+^ T-cells and Proliferating (Ki67^+^) CD4^+^ T-cells. **d**. tSNE visualizations showing CD4^+^ T-cell subpopulations by clinical group. **e**. Stacked bar plot representing cell subpopulation proportions within individual patients grouped by NR, TCMR, and CR. **f.** Boxplots showing CD4^+^ cell subpopulation percent per patient as a fraction of the CD4+ T-cell population. Resident memory T-cells represented the most abundant phenotype observed in NR; CD3^+^CD4^+^ T-cells was the predominant phenotype detected in both TCMR and CR groups, which presented a greater per proportion of activated CD4^+^ T-cells; TCMR showed a greater proportion of Naïve CD4^+^ and PD1^+^ T-cells as well as HLADR^+^ T-regs compared to NR. Overall P-values are based on the Kruskal-Wallis test and pairwise comparisons across clinical group are based on the Wilcoxon rank sum test and corrected for multiple tests. **g**. Pseudotemporal trajectory analysis of the CD4^+^ compartment with UMAP of cell populations. The leftmost panel shows UMAP plot with cell subpopulations and second panel shows the predicted temporal trajectory (black line, bottom to top). The rightmost panel depicts the density of CD4+ T-cells (y-axis) from each clinical group across Pseudotime (x-axis). PD1^+^CD4^+^ T-cells and Tregs represent a late-stage of differentiation of effector CD4^+^ T-cells specific of TCMR.

D4 + T-cells: Within the CD4^+^ T-cell compartment, nine total subclusters were identified (Fig. 2, **Extended Data** Fig. 3a). We initially stratified CD4^+^ T-cells by CD3 expression, resulting in a CD3^Low^CD4^+^ T-cell subset and a CD3^High^CD4^+^ T-cell subset. Variations in CD3 expression within the CD4^+^ T-cell compartment have been described, with low levels corresponding to resident memory CD4^+^ T-cells and high levels associated with an activated state^39,40^. The resident memory CD4^+^ T-cell subset was more abundant in NR, while CD3^High^CD4^+^ T-cells were more abundant in TCMR and CR (Fig. 2d–f, **Extended Data** Fig. 3b-c). Compared to NR, TCMR had a greater proportion of CD3^high^CD4^+^ T-cells, naïve CD4^+^ T-cells, and activated CD4^+^ T-cells, which is consistent with acute alloreactivity (Fig. 2f). While their overall frequency was rare, we observed a concomitant increase in regulatory cell types, including HLADR^+^ Tregs and PD1^+^ CD4^+^ T-cells, in the TCMR group when compared to NR, suggesting that their expansion counters effector alloreactive T-cell activity (Fig. 2f). We also determined that the CD3^high^CD4^+^ T-cell subset represented most of the CD4^+^ T-cells in CR, with a significant decrease in resident memory T-cells and higher proportion of activated T-cells when compared to NR (Fig. 2f). Unlike TCMR, there was no expansion of the regulatory HLADR^+^ Treg or PD1^+^ CD4^+^ T-cell populations in CR. To understand the lineage maturation trajectory of CD4^+^ T-cells in the alloimmune microenvironment, pseudotime reconstruction was performed (Fig. 2g)^32^. This provides further evidence that NR is primarily associated with CD4^+^ resident memory T-cells and suggests that CD4^+^ T-cell subpopulations increased during TCMR and CR originate and proliferate from circulating CD4^+^ T-cells (Fig. 2g). These data also suggest that the expanded Treg and PD1 + CD4 + T-cells observed in TCMR represent late-stage effector cells unique to this phase of alloimmunity.

**Figure 3:**
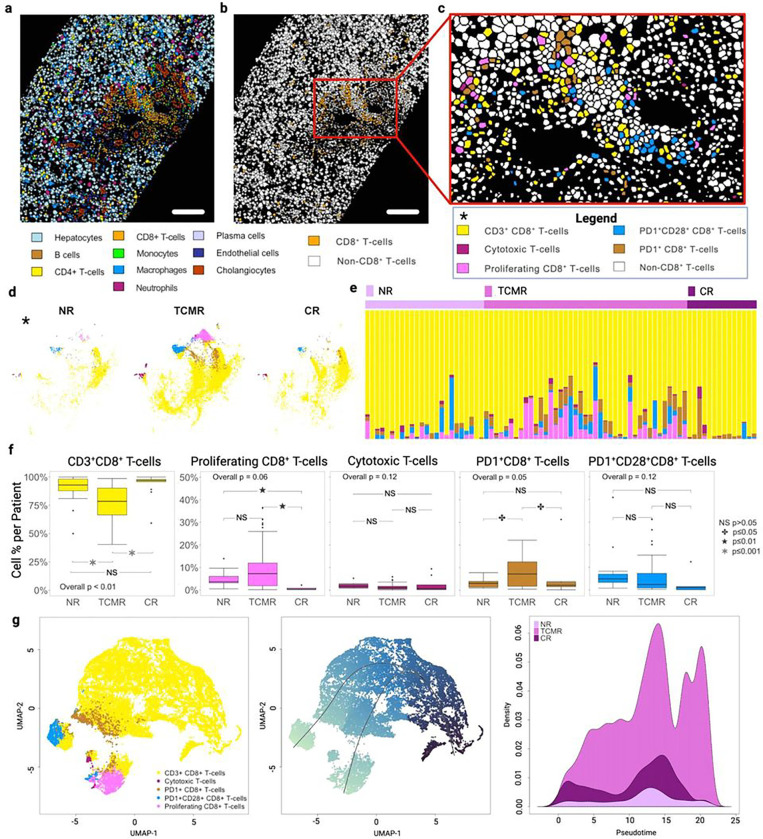
CD8^+^ T-cell profile in TCMR highlights simultaneous increases in cell proliferation and signs of exhaustion phenotype. **a.** Representative TCMR image with metaclusters projected onto the mask outline of core biopsy region of interest (scale bar = 190mm). **b**. TCMR mask image now highlighting CD8^+^ T-cells only in orange. **c.** Zoom panel of CD8^+^ T-cells colored by cell subpopulation (see color key). Similar to with CD4+ T-cells, the CD8^+^ compartment was categorized into subpopulations using unsupervised clustering with the following markers: CD28, CD16, CD11b, CD45, CD8, CD279 (PD1), FoxP3, Ki67, CD3, HLADR, and Granzyme B. Five unique subpopulations were identified from the parent CD8+ population comprising 30,488 total cells: CD3^+^CD8^+^ T-cells, Proliferating (Ki67^+^) T-cells, Cytotoxic T-cells, PD1^+^CD8^+^ T-cells, PD1^+^CD28^+^ T-cells. **d**. tSNE of CD8^+^ T-cell subpopulations. **e.** Stacked bar plot showing individual CD8^+^ T-cell subpopulations by patient and clinical group. **f**. Boxplots depicting CD8^+^ T-cell subpopulations as a percent of total CD8^+^ T-cell population and compared across clinical group with Overall P-value calculated using the Kruskal-Wallis test and pairwise P-values from the Wilcoxon rank sum test, corrected for multiple comparisons. Different distribution in CD3^+^CD8^+^ T-cells, proliferating and PD1^+^CD8^+^ T-cells subpopulation was observed across the three clinical groups, with a greater proportion of proliferating and PD1^+^CD8^+^ T-cells in TCMR **g**. Leftmost panel with Pseudotime UMAP plot of CD8^+^ T-cell subpopulations and middle panel with predicted trajectory showing dual trajectory starting at the darker portion of the graph and moving to the lower left of the plot. Plot of density of CD8^+^ T-cells (y-axis) in each clinical group across Pseudotime (x-axis). Stimulation of CD3^+^CD8^+^ T-cells result in the maturation of two distinct phenotypes represented by a proliferating CD8^+^ T-cells and a PD1^+^CD8^+^ T-cells subpopulations.

CD8 + T-cells: Subclustering of the CD8^+^ T-cell compartment identified five different CD8 + T-cell subsets, including CD45^+^CD3^+^CD8^+^ (‘CD3^+^CD8^+^ T-cells’), Ki67^+^CD45^+^CD3^+^CD8^+^ (‘Proliferating CD8^+^ T-cells’), GranzymeB^+^CD45^+^CD3^+^CD8^+^ (‘Cytotoxic T-cells’), PD1^+^CD45^+^CD3^+^CD8^+^ (‘PD1^+^CD8^+^ T-cells’), and PD1^+^CD28^+^CD45^+^CD3^+^CD8^+^ (‘PD1^+^CD28^+^CD8^+^ T-cells’) (Fig. 3a–c, **Extended Data** Fig. 3d). Even though the overall CD8 + T-cell proportion differed between clinical groups (Fig. 1e), the CD3^+^CD8^+^ T-cell subgroup was most abundant subset in each cohort (Fig. 3d–e). TCMR showed a higher overall frequency of CD8 + T-cells when compared to both NR and CR (p < 0.01, Fig. 1e), and this was predominately related to an increased frequency of proliferating CD8^+^ T-cells, supporting the concept of effector CD8 + T-cell expansion during acute alloimmunity (Fig. 3d–f, **Extended Data** Fig. 3e). Cytotoxic T-cells were rare and showed no differences across clinical groups within the CD8^+^ T-cell compartment (Fig. 3f, **Extended Data** Fig. 3e). Similar to what was observed for CD4^+^ T-cells, TCMR tissue exhibited a greater enrichment of PD1^+^CD8^+^ T-cells when compared to NR and CR (p < 0.05) (Fig. 3e–f). Pseudotime analysis to evaluate CD8^+^ T-cell lineage maturation revealed two divergent paths of the abundant CD3^+^CD8^+^ T-cells, into either the PD1^+^ phenotype or the Ki67^+^ proliferating CD8^+^ phenotype (Fig. 3g). This suggests that this population has is its own terminally differentiated function and is not merely a cell subpopulation displaying exhaustion markers post proliferation.

**Figure 4:**
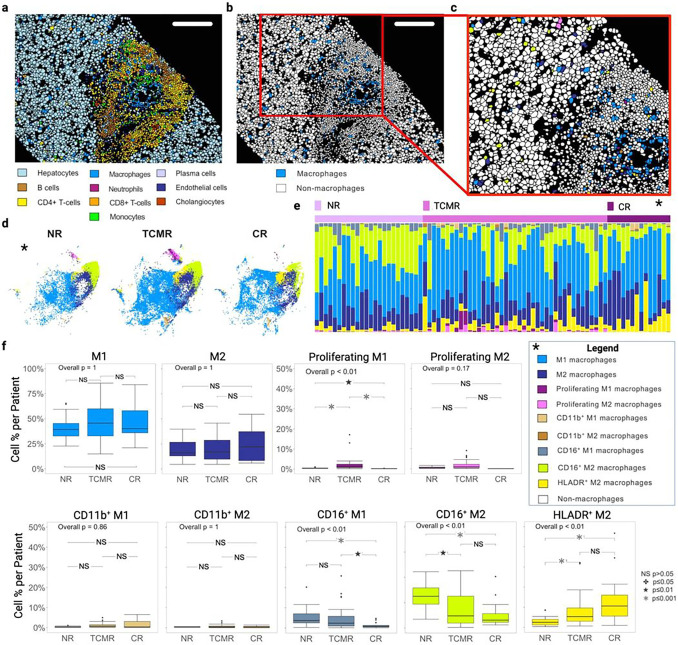
Both TCMR and CR are characterized by increased proportion of HLADR+ M2 macrophages with concurrent decreases in CD16^+^ M1 and M2 macrophages. **a**. Cell mask visualization on TCMR tissue section colored by metacluster (scale bar = 190mm). **b**. TCMR tissue section again with cell mask outlines and colored blue to show location of the macrophage metacluster cells within the tissue. **c**. Zoom panel of macrophage subpopulations (see color key legend). The macrophage metacluster was comprised of 45,927 total cells within the entire dataset and subpopulations were identified by first differentiating M1 (CD163^Lo^) from M2 (CD163^hi^) then performing unsupervised clustering based on expression of CD16, CD11b, CD45, FoxP3, CD163, CD68, Ki67, and HLADR. Nine distinct subpopulations emerged from this analysis including generic M1 and M2 populations, Proliferating (Ki67^+^) M1 macrophages, Proliferating (Ki67^+^) M2 macrophages, CD11b^+^ M1 macrophages, CD11b^+^ M2 macrophages, CD16^+^ M1 macrophages, CD16^+^ M2 macrophages and HLADR^+^ M2 macrophages. **d**. tSNE plot of macrophage subpopulations separated by clinical group. **e**. Stacked bar plot of individual macrophage subpopulations by patient and clinical group. **f**. Boxplots showing macrophage subpopulations as a percent of the overall macrophage population per patient with overall P-value derived from the Kruskal-Wallis test. Pairwise analysis from Wilcoxon rank sum test corrected for multiple comparisons. A greater proportion of proliferating M1 macrophages was observed in TCMR compared to NR and CR; TCMR and NR had a greater proportion of CD16^+^M1 macrophages compared to CR; NR showed a greater cell percentage of CD16^+^ M2 macrophages compared to TCMR and CR; HLADR^+^ M2 macrophages were more abundant in both TCMR and CR compared to NR.

Macrophages: Among the immune meta-clusters, macrophages were the most abundant cell type (**Extended Data** Fig. 1d) in all clinical groups, which highlights their key role in liver homeostasis, disease, and injury processes^37,38^. Indeed, macrophages can participate in robust infiltration of the AME during severe rejection episodes; however, their role has rarely been investigated in TCMR and CR in clinical LT^24,41^. We have previously shown that CR is characterized by a discrete macrophage phenotype absent in NR^24^. Thus, to obtain a detailed representation of the macrophages complexity and heterogenous activity in LT, we first divided macrophages M1 and M2 based on their expression of CD163 (**Extended Data** Fig. 4a)^42^. The overall distribution of M1 and M2 did not differ among NR, TCMR, and CR, nor did the ratio of M2:M1 macrophages (**Extended Data** Fig. 4b-d)^42^. Subclustering revealed four M1 (‘M1’ (CD68^+^CD163^Lo^) ‘CD11b + M1’ (CD11b^+^CD68^+^CD163^Lo^), CD16 + M1 (CD16^+^CD68^+^CD163^Lo^), and ’proliferating M1’ (Ki67^+^CD68^+^CD163^Lo^)) and five M2 macrophage subtypes (‘M2’ (CD68^+^CD163^Hi^) ‘CD11b + M2’ (CD11b^+^CD68^+^CD163^Hi^), CD16 + M2 (CD16^+^CD68^+^CD163^Hi^), ‘proliferating M2’ (Ki67^+^CD68^+^CD163^Hi^) and ‘HLADR^+^ M2’ (HLADR^+^CD68^+^CD163^Hi^), Fig. 4a–c, **Extended Data** Fig. 4e). Consistent with the activation of an inflammatory process, a greater percentage of proliferating M1 macrophages was observed in TCMR compared to NR and CR (Fig. 4f). We found one M1 and one M2 macrophage subset each expressing CD16. Both NR and TCMR exhibited a greater percentage of CD16^+^ M1 macrophages when compared with CR. The CD16^+^ M2 macrophage subcluster was most abundant in NR and appeared to become progressively depleted from TCMR to CR. These cells might represent a population of regulatory and anti-inflammatory macrophages (M2b), capable of IL-10 secretion (Fig. 4f)^43,44^. A subpopulation of HLADR^+^ M2 macrophages showed the opposing pattern to CD16^+^ M2 cells and was more abundant in both TCMR and CR than NR (Fig. 4f). These HLADR^+^ M2 macrophages might represent a different activation state compared with the generic M2 macrophage subpopulations or suggest a unique specialization of those cells such as for antigen presentation.

Monocytes: Within the monocyte metacluster, which contained only 1.4% of all cells identified in AME, we defined four cell phenotypes: classical (CD11b^+^CD16^+^), non-classical (CD11b^+^CD16^−^), intermediate (CD11b^+^CD16^+^HLADR^+^), and activated monocytes (CD11b^+^CD16^−^HLADR^+^, **Extended Data** Fig. 5a). On tissue visualization, both classical and activated monocytes were spatially located next to blood vessels, while non classical monocytes resided at distance from vascular structures (**Extended Data** Fig. 5b-d). Classical monocytes represented the most abundant subset across all clinical groups (**Extended Data** Fig. 5e-f), and the comparison of percentage across the three allograft states showed that intermediate and non-classical monocytes comprised a greater proportion of the monocyte metacluster in NR compared with TCMR and CR (**Extended Data** Fig. 5g).

B-cells and Plasma cells: B cells represented the smallest metacluster in the overall dataset (4,881 or 1% of all cells identified, Fig. 1c). Comparison of the three B cell subpopulations identified did not highlight any difference across different alloimmune states (**Extended data** Fig. 6). Finally, the small fraction of plasma cells identified, approximately 1.2% of all cells contained in the dataset, showed a higher proportion of those cells in TCMR than CR (Fig. 1e).

### Spatial interaction and multicellular functional motifs define liver allograft rejection pathology

**Figure 5:**
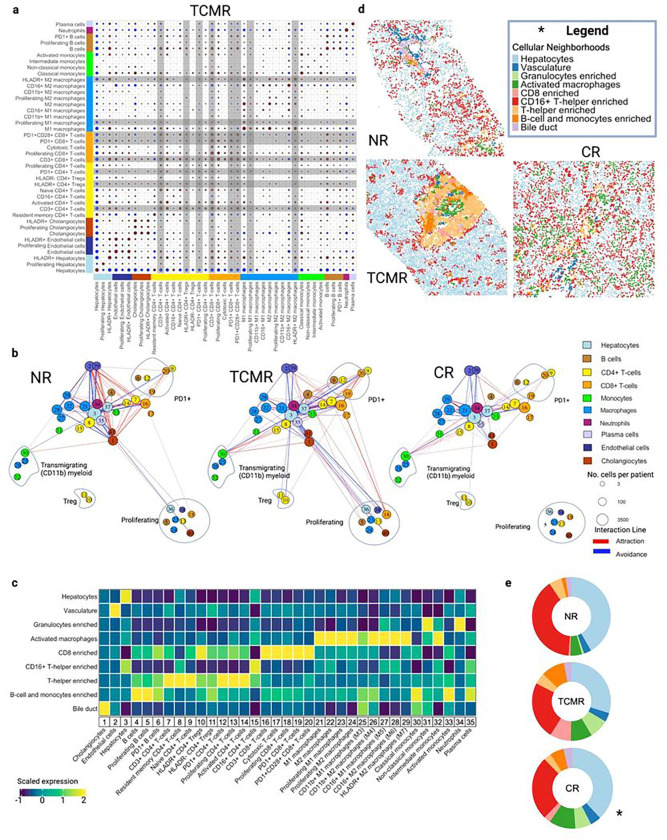
Spatial profiling of liver allograft biopsies uncovers 8 cellular neighborhood motifs that are differentially abundant across clinical groups. **a**. Heatmap showing pairwise spatial interaction between subclusters in TCMR. **b**. Spatial correlation network visualization showing attractions (red line) and avoidances (blue line) across cell subpopulations and colored by the metacluster that the subpopulation is derived from. The line thickness represents the strength of the degree of attraction or avoidance between the cell subpopulations and the size of the circle represents the size of the subpopulation. For ease of visualization, the CD11b+ monocyte/macrophage, PD1+, Proliferating and Treg populations are grouped in phenotype clusters (grey circle highlights). Numbering of the subclusters otherwise corresponds to labels from the heatmap in part c (including the following: cluster 36=proliferating hepatocytes, 37=HLADR+ hepatocytes, 38=proliferating endothelial cells, 39=HLADR+ endothelial cells, 40=proliferating cholagiocytes, 41=HLADR+ cholangiocytes). Lymphocytes exhibiting an exhausted phenotype (clusters number 6, 12, 19, 20) showed a greater number of interactions in TCMR compared to NR and CR. **c**. Heatmap showing composition of cellular neighborhood (CN) clusters. From the 35 identified cell populations and subpopulations in our dataset, we obtained 9 distinct cellular neighborhoods or spatial motifs that are found within our dataset which include: Hepatocyte, vasculature, granulocyte enriched, activated macrophages, CD8 enriched, CD16+ T-helper enriched, T-helper enriched, B cell and monocyte enriched, and bile duct. **d**. Visualization of cellular neighborhoods projected onto representative biopsy specimens from NR, TCMR and CR. **e**. Donut plots showing proportions of cellular neighborhoods by clinical group. TCMR has the largest proportion of CD8 enriched and B cell and monocyte enriched cellular neighborhoods. NR has the proportion of CD16+ T-helper enriched CN.

Next, we examined the spatial data layer from our single cell proteomic IMC atlas to assess pairwise relationships between immune subpopulations within each clinical group by applying neighborhood and correlation analysis to characterize the statistical probabilities of cell-cell interactions (Fig. 5). Overall, a greater number of interactions, either via avoidance or attraction, were observed in TCMR when compared to NR and CR (Fig. 5a–b, **Extended Data** Fig. 7a-b). The AME in TCMR was characterized by CD3^+^CD8^+^ T-cells showing attraction to APCs including proliferating and M1 macrophages, classical monocytes, HLADR^+^ M2 macrophages and B-cells, as well as CD3^+^CD4^+^ T-cells supporting the idea of complex multicellular interactions characterizing this pro-inflammatory state (Fig. 5a). The neighborhood analysis revealed the presence of exhausted T-cells (PD1^+^CD4^+^, PD1^+^CD28^+^CD8^+^, and PD1^+^CD8^+^ T-cells) and Tregs in the vicinity of effector T-cells, which established a greater number of positive interactions when compared to NR, suggesting that a close crosstalk between those two ends of the spectrum T-cell phenotypes occurs in TCMR (Fig. 5a–b, **Extended Data** Fig. 7a). Conversely, resident memory CD4^+^ T-cells showed no contact or avoidance with exhausted phenotypes in NR and TCMR respectively (Fig. 5a–b, **Extended Data** Fig. 7a). In CR, HLADR^+^ M2 macrophages surrounded HLADR + hepatocytes and M1 macrophages, while reciprocal strong interaction between CD16^+^ M2 macrophages and M2 macrophages were observed, likely representing a niche in which further differentiation of M2 macrophages occurs (**Extended Data** Fig. 7b).

Due to the relationship of the RAI score for TCMR to endothelial inflammation, we evaluated the distributions of distance to endothelial cells and each immune subpopulation across clinical groups (**Extended Data** Fig. 7c). Most pro-inflammatory subpopulations including CD3^+^CD4^+^ and CD3^+^CD8^+^ T-cells, proliferating and cytotoxic CD8 + T-cells, and classical monocytes resided near CD31^+^ endothelial cells, while resident memory CD4 + T-cells, CD16^+^ M1 macrophages, and CD16^+^ M2 macrophages were distributed throughout the tissue (**Extended Data** Fig. 7c).

To evaluate higher order spatial motifs as potential functional units associated with liver allograft pathology, we performed cellular neighborhood analysis, where patterns of higher order cellular structures (with 10 nearest neighbors) were clustered into nine novel cellular neighborhoods (CNs). These were labeled according to the cell types in each cluster as shown in the heatmap in Fig. 5c: hepatocytes, vasculature, granulocyte enriched, activated macrophages, CD8 enriched, CD16^+^ T-helper enriched, T-helper enriched, B-cell and monocyte enriched, and bile ducts (Fig. 5c–d). We then visualized and compared the proportions of these CNs across clinical groups (Fig. 5d–e, **Extended Data** Fig. 7d). For the non-immune predominant CNs (hepatocyte, vascular, and bile duct) there were few differences between clinical groups except for a slightly smaller proportion of the hepatocyte CN in TCMR (likely a direct consequence of the increase of immune cell enriched CNs in TCMR, **Extended Data** Fig. 7d). The CD8 enriched CN (which included HLADR + CD4 + Treg and both PD1^+^CD8^+^ T-cell subpopulations) was expanded in TCMR when compared with NR and CR (p < 0.001 and p < 0.01 respectively). Additionally, the ‘B cell and monocyte’ CN was most abundant in TCMR and was also expanded in CR compared to NR (p < 0.001). Conversely, the CD16^+^ T-helper enriched CN was more abundant in NR when compared with TCMR and CR (p < 0.001), suggesting that this CN could be a marker of allograft tissue homeostasis (**Extended Data** Fig. 7d).

### Evaluation of exhaustion markers in liver biopsy tissue via RNA Sequencing

**Figure 6:**
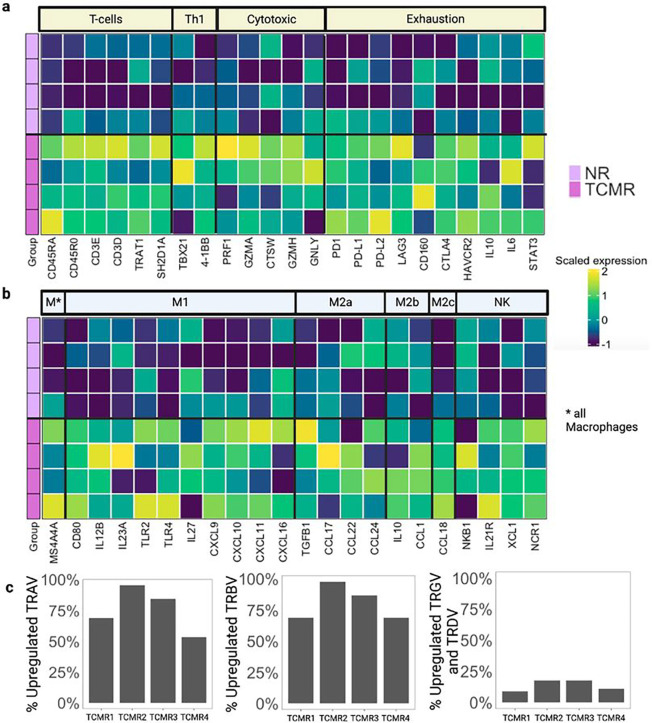
In depth molecular characterization of tissue using bulk RNA sequencing (nCounter) panel confirms a mix of phenotypes enriched in T-cells and macrophages in TCMR. **a**. T-cell populations predicted from a set of 4 NR tissue specimens used in our dataset and 4 TCMR tissue specimens. Genes correspond to generic T-cells, Th1, cytotoxic and exhausted phenotypes. Activated, cytotoxic as well as exhausted T-cell genes showed a greater expression in TCMR compared to NR. **b.** Heatmap of scaled expression values of macrophage and NK-related genes including M1, M2a, M2b, M2c phenotypes along with NK-associated genes. Genes belonging to both M1 and M2 polarized macrophages showed a greater expression in TCMR than NR; similarly, NK-associated genes were upregulated in TCMR. **c**. Histograms showing percentages of upregulated TCR alpha (TRAV) genes, TCR beta (TRBV), and TCR gamma delta (TRGV and TRDV) genes respectively across samples. The increased expression of different TCR alpha, beta and gamma-delta associated with TCMR, is in agreement with expansion in the number of T-cell clones.

Our single cell atlas across the spectrum of rejection in LT identified several unique cell types increased during TCMR, including Tregs, PD1 + CD4 + T-cells, PD1 + CD8 + T-cells, and HLADR + M2 macrophages. To obtain further evidence for the functional status of these populations, including whether the identified PD1^+^ T-cells represent a terminally differentiated, activated CD4^+^ or CD8^+^ T-cell versus an exhausted effector T-cell population, we performed bulk transcriptomic analysis using the nCounter platform^45^. Liver core biopsies from 8 samples, comprising the most representative 4 TCMR and 4 NR cases were used for analysis. We selected 23 genes defining T-cell phenotypes including helper function, exhaustion, and cytotoxic activity (Fig. 6a). By comparing DE genes between NR and TCMR, we identified an overall upregulation of genes typically associated with cytotoxic activity and as well as upregulation of PDCD1 (Programmed cell death 1 or PD1) gene expression in TCMR samples, which is consistent with the higher percentage of PD1^+^CD4^+^ and CD8^+^ T-cell identified in our IMC dataset (Fig. 6a). We also observed increased DE of PDCD1LG1 (Programmed cell death-ligand 1 or PD-L1), PDCD1LG2 (Programmed cell death-ligand 2 or PD-L2), CTLA4 (Cytotoxic T-Lymphocyte Associated Protein 4), Lag3 (Lymphocyte activating 3), and CD160 (CD160 antigen) genes in TCMR compared to NR, confirming upregulation of both ligands for PD1 and T-cell exhaustion markers (Fig. 6a). We selected 18 genes which differentiate the diverse macrophage polarization in M1, M2a, M2b and M2c (Fig. 6b). Genes for both pro-inflammatory cytokines such as CXCL9 (C-X-C motif chemokine ligand 9) and CXCL10 (C-X-C motif chemokine ligand 10), mainly expressed by M1 macrophages, and anti-inflammatory cytokines including IL-10 (Interleukin 10), CCL22 (C-C motif Chemokine Ligand 22), CCL24 (C-C motif Chemokine Ligand 24) mostly associated with M2 macrophage polarization, were upregulated in TCMR when compared to NR. We have not yet identified a reliable NK marker for IMC in liver tissue, so we also evaluated markers of NK cells including IL21R (Interleukin 21 receptor), XCL1 (X-C motif Chemokine Ligand 1) and NCR1 (natural cytotoxicity triggering receptor 1), which were upregulated in TCMR samples (Fig. 6b). We also identified an overall upregulation of several genes associated with neutrophils, B cells, mast cells, and dendritic cells (**Extended data Fig**. 8a). We validated these findings using a publicly available database including of 6 cases of biopsy-proven TCMR in clinical LT, demonstrating upregulation of the same DE genes from our analysis (**Extended data Fig. 8b-c**)^34^.

Finally, we evaluated the intragraft T-cell receptor (TCR) repertoire to determine whether alloreactive T-cells during TCMR exhibited oligoclonal expansion via bulk analysis of TCR diversity using the 129 gene TCR Diversity nCounter assay. By using the average expression of TCR genes in NR samples as baseline, we analyzed which TCR variable region genes were upregulated or downregulated in TCMR compared to NR. We found an upregulation in the majority of TCR alpha (TRAV), TCR beta (TRBV), and TCR delta (TRDV) and TCR gamma (TRGV) variable region genes (Fig. 6c). These data suggest that TCMR is associated with an increase in the number of T-cells clones rather than expansion of few pathogenic alloreactive clones, which is similar to prior reports^46^.

### Cell composition is highly predictive of rejection in liver allografts

**Figure 7:**
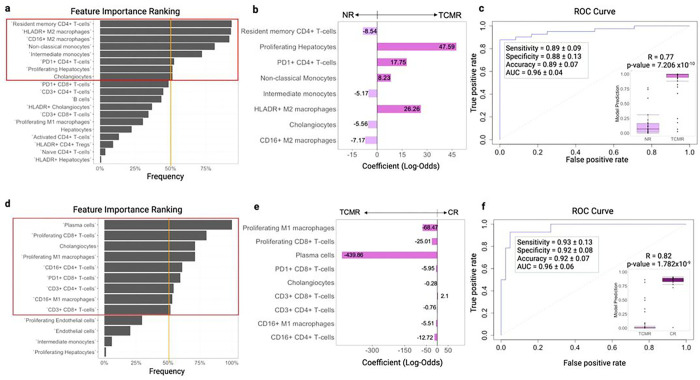
Indentification of cellular features of liver allograft biopsies are highly predictive of discriminating TCMR from NR and CR. **a**. Bootstrapping using logistic LASSO regression identified the top highly ranked features which are predictive of NR vs TCMR. **b**. Based on the model, identified subpopulations which are well suited for distinguishing NR from TCMR are resident memory CD4+ T-cells, proliferating hepatocytes, PD1+ CD4+ T-cells, non-classical monocytes, intermediate monocytes, HLADR+ M2 macrophages, cholangiocytes, CD16+ M2 macrophages. Positive coefficient indicates that an increase of that cell subpopulation increases the likelihood of TCMR, while negative coefficient indicates that an increase of that cell subpopulation decreases the likelihood of TCMR, thus increasing the likelihood of NR. **c**. Evaluation metrics for predictive model built using highly ranked cell subpopulations identified in a. The model shows a sensitivity of 0.89 ± 0.09, specificity of 0.88 ± 0.13, accuracy of 0.89 ± 0.07, and area under the curve (AUC) of 0.96 ± 0.04 (mean ± SD). Spearman correlation coefficient between median predicted and actual outcomes $R=0.77;\;p-value\;=7.206\times 10^{-10}$ (Wilcoxon rank-sum test). **d**. Bootstrapping using logistic LASSO regression model identified the top highly ranked features which are predictive of TCMR vs CR. **e**. Based on the model, identified subpopulations which are well suited for distinguishing TCMR from CR are proliferating M1 macrophages, Proliferating CD8+ T-cells, Plasma cells, PD1+ CD8+ T-cells, cholangiocytes, CD3+CD8+ T-cells, CD3+CD4+ T-cells, CD16+ M1 macrophages, CD16+ CD4+ T-cells. Positive coefficient indicates that an increase of that cell subpopulation increases the likelihood of CR, while negative coefficient indicates that an increase of that cell subpopulation decreases the likelihood of CR, thus increasing the likelihood of TCMR. **f.** Evaluation metrics for predictive model built using highly ranked cell subpopulations identified in d. The model shows a sensitivity of 0.93 ± 0.13, specificity of 0.92 ± 0.08, accuracy of 0.92 ± 0.07, and area under the curve (AUC) of 0.96 ± 0.06 (mean ± SD). Spearman correlation coefficient between median predicted and actual outcomes $R=0.82;\;p-value\;=1.7827\times 10^{-9}$ (Wilcoxon rank-sum test).

The analysis of the AME in NR, TCMR and CR identified 41 potential features of which 14 immune and 5 non-immune differed in patients who developed TCMR from NR, 9 immune and 4 non-immune features distinguished TCMR from CR, and 10 immune and 1 non-immune features separated out NR from CR, thereby highlighting complex network of different cell phenotypes specific for those three AMEs. To determine whether these immune phenotypes could predict patient outcomes, the logistic LASSO regression algorithm was applied to the entire dataset. To improve model robustness, 5-fold cross-validation was used to determine model parameters, and a 5,000 iterative bootstrapping technique was used to perform feature selection by determining feature importance based on frequency. To ensure that predicted results and model performance are derived from patients not included when training the model, a 1,000 iterative random training/validation split was implemented; performance metrics were averaged and the median prediction of each patient was used for further model evaluation. Examination of the most important features, which present a frequency ≥ 50%, revealed that 8 cell subpopulations contributed the most in generating a model that can accurately differentiate TCMR vs NR (mean ± SD; accuracy 0.89 ± 0.07 and mean area under the curve (AUC) 0.96 ± 0.04) demonstrating a high correlation between median prediction and actual clinical outcome (Spearman correlation coefficient $R=0.77,\;p=7.206\times 10^{-10}$) (Fig. 7a,c **and Extended Data Fig. 9a**). The highest-ranking immune phenotype was resident memory CD4^+^ T-cells as a predictor of NR, corresponding to pairwise analysis demonstrating that this immune subset was strongly associated with NR (Fig. 2). In addition, intermediate monocytes, cholangiocytes, and CD16^+^ M2 macrophages were predictors of NR, whereas PD1^+^CD4^+^ T-cells, HLADR^+^ M2 macrophages, non-classical monocytes, and proliferating hepatocytes were positive predictors of TCMR (Fig. 7b). Application of this modelling approach to differentiate TCMR from CR resulted in 9 highly ranked features that can accurately distinguish these two alloimmune states, with a high sensitivity, specificity, and accuracy, and a mean AUC of 0.96 ± 0.04 (Spearman correlation coefficient between prediction and actual outcome of 0.82, $p=1.782\times 10^{-9}$) (Fig. 7d,f). Among these features, proliferating and CD16^+^ M1 macrophages, proliferating and PD1^+^ CD8 + T-cells, plasma cells, CD3^+^ and CD16^+^ CD4 + T-cells predicted TCMR while the CD3^+^CD8^+^ T-cell phenotype was a predictor of CR (Fig. 7e **and Extended Data Fig. 9b**). Modelling for differentiating NR from CR was also highly sensitive and specific, however the sample size for this comparison (38 samples total) may be too small to generate a conclusion (**Extended Data Fig. 9**). Overall, these results indicate that rare but specific cell subpopulations identified in the present study can potentially harbor high diagnostic value in biopsies obtained across the spectrum of rejection in clinical LT.

## DISCUSSION

Our study provides a comprehensive single cell, spatially resolved analysis of the AME in clinical LT, revealing the complexity of alloimmunity in solid organ transplant recipients. Unlike the cancer tumor microenvironment, which has remarkable phenotypic variability between patients and even within the same specimen, our analysis confirms central tenants of transplant immunology, namely that the pathologic features within the AME are similar across individuals, despite differences in patient demographics, underlying etiology of liver disease, features of the donor organ, and timing of rejection episodes. Thus, study of the AME offers an ideal application and proof-of-concept for further development of spatial proteomics immunologic analyses using archival biopsy specimens. Further, exploration of discrete immune subpopulations within the AME of core needle liver biopsies has identified immune subsets with exhaustion phenotypes that are enriched in TCMR and largely absent in CR, providing new insights into the mechanistic underpinnings and evolution of liver allograft rejection. Finally, these data were harnessed to create a predictive model of TCMR and CR using a subset of cell types, which offers potential for clinical use to diagnose rejection states more accurately when compared to the current standard of care that relies on subjective pathologic evaluation using the RAI.

Single cell analysis of the AME has uncovered substantial complexity in allograft rejection, involving at least 32 distinct immune subpopulations. Nearly all prior studies of LT rejection have focused on one or few immune cell types^47,48^. In clinical TCMR, our data demonstrate that diverse cell populations contribute to the underlying pathophysiology. It is increasingly recognized that spatial context is important to completely describe disease phenotypes and that these multiplexed spatial techniques will have critical clinical implications^49^. A recent study in LT recipients examined immune cell type pairs at high resolution to evaluate immune synapse formation and used these data to predict success of immunosuppression withdrawal^11^. These data, taken together with our results, suggest that further characterization of important features of the AME will provide valuable insights into predicting clinical outcomes with greater precision than is currently possible.

Arising from the complex microenvironment was a central theme of immune exhaustion. Our study design captures clinical specimens prior to initiation of rejection treatment, the mainstay of which are calcineurin inhibitors (CNI), which are designed to indiscriminately prevent T-cell proliferation and come with potentially severe side effects. Fortunately, the immune system has physiologic mechanisms to dampen this immune response through PD-1 ligand, a molecule that when knocked out in mice results in auto-immunity, and is also important in chronic inflammatory states^15,50,51^. Our results show PD1^+^CD4^+^ T-cells and PD1^+^CD8^+^ T-cells are expanded in TCMR and spatially interacting with other CD4^+^ and CD8^+^ subpopulations, as well as M2^+^HLADR^+^ macrophages. Predictive modelling classified PD1 + CD4 + T-cells as a feature distinguishing TCMR from NR, while PD1 + CD8 + T-cells were identified as a feature distinguishing TCMR from CR. Taken together, these data indicate that immune exhaustion may be a key mediator of TCMR in the AME.

Dysregulated exhaustion states are increasingly recognized as a pathway cancer cells manipulate to mediate immune escape, leading to development of immune checkpoint inhibitor therapies to enhance anti-tumor adaptive immune responses. Our data suggest the opposite therapeutic approach should be explored to counteract pro-inflammatory responses during acute TCMR, via augmentation of physiologic exhaustion. Indeed, prior work using PD-1 agonists in other pro-inflammatory states, including neutrophilic asthma, has demonstrated that therapies designed to promote T-cell exhaustion can mitigate inflammation^16^.

For the past 60 years, pathologic detection of allograft rejection has been conducted using Hematoxylin and Eosin staining. The Banff RAI is then used to characterize rejection by evaluation of portal and/or perivenular inflammatory (immune) infiltrates ^52^. Our study suggests that spatial relationships between immune cells and cholangiocytes or endothelial cells may be less important mechanistically. Rather, investigation into the presence or absence of certain immune subpopulations may better inform important considerations of TCMR such as steroid-resistant disease, a disease that often progresses to CR and drives late graft failure. Predictive modelling combined with IMC may bring value in this regard. A recent study used IMC combined with deep learning to predict lung adenocarcinoma progression and patient survival post-surgery with high accuracy^53^. Harnessing multiplexed data together with emerging artificial intellegnce tools such as deep learning may have profound diagnostic and prognostic value, both in clinical practice and to monitoring responses to treatment in clinical trials. A major benefit of IMC over transcriptomic platforms is that IMC can be employed on archival FFPE samples from a small core biopsy without concern for RNA degradation. Furthermore, proteomics-based platforms like IMC more accurately reflect single cell phenotypes given that RNA is not always linearly correlated with protein translation^54^. In our study, clinically diagnostic areas selected from core biopsies resulted in over 5000 cells per ROI, which is comparable to cell counts obtained from scRNA seq experiments in human liver, without bias caused by pre-analytical variables, including cellular damage and loss, inherent to tissue dissociation and processing techniques necessary to create a single cell suspension^55^.

Our analysis is limited by the inability to conduct complementary transcriptomics or cell-culture based assays as our study was performed on a retrospective set of tissue formalin-fixed and paraffin embedded specimens collected during routine clinical care. Furthermore, there is the possibility of cell classification errors within our IMC dataset, particularly within the hepatocyte metacluster that was classified based on exclusion of other cell types. To minimize this risk, we conducted an extensive review all annotated tissue specimens alongside raw marker expressions to ensure that tissue labelling was optimized. Due to the unexpected importance of exhaustion phenotypes in our results, our IMC dataset lacked PD-L1 and other exhaustion markers, and these will be incorporated for future studies. Finally, our patient sample size was somewhat limiting for our results, particularly with predictive modelling in CR; however, our analyses support our study being adequately powered for the evaluation of important cell subpopulations and in the modelling of TCMR.

Herein, we provide a novel, detailed, and spatially resolved atlas of clinical liver allograft rejection. Highly multiplexed IMC-based analyses uncovered unique features of the AME and predictive features of rejection states. We further identified immune exhaustion as a central feature of TCMR, suggesting the PD-1 pathway as a potentially novel therapeutic target in liver allograft rejection. This work provides a conceptual framework for investigation of inflammatory processes in immunologically complex histological diseases of the liver using clinical samples.
